# Nanocrystalline Lanthanum Oxide Layers on Tubes Synthesized Using the Metalorganic Chemical Vapor Deposition Technique

**DOI:** 10.3390/ma17225539

**Published:** 2024-11-13

**Authors:** Agata Sawka

**Affiliations:** Faculty of Materials Science and Ceramics, AGH University of Krakow, al. A. Mickiewicza 30, 30-059 Krakow, Poland; asawka@agh.edu.pl

**Keywords:** lanthanum oxide layers, MOCVD process, tubular substrates, UV-Vis spectroscopy

## Abstract

Lanthanum oxide (La_2_O_3_) layers are widely used in electronics, optics, and optoelectronics due to their properties. Lanthanum oxide is also used as a dopant, modifying and improving the properties of other materials in the form of layers, as well as having a large volume. In this work, lanthanum oxide layers were obtained using MOCVD (Metalorganic Chemical Vapor Deposition) on the inner walls of tubular substrates at 600–750 °C. The basic reactant was La(tmhd)_3_ (tris(2,2,6,6-tetramethyl-3,5-heptanedionato)lanthanum(III)). The evaporation temperature of La(tmhd)_3_ amounted to 170–200 °C. Pure argon (99.9999%) and air were used as the carrier gases. The air was also intended to remove the carbon from the synthesized layers. Tubes of quartz glass were used as the substrates. La_2_O_3_ layers were found to be growing on their inner surfaces. The value of the extended Gr_x_/Re_x_^2^ criterion, where Gr—Grashof’s number, Re—Reynolds’ number, x—the distance from the gas inflow point, was below 0.01. The microstructure of the deposited layers of lanthanum oxide was investigated using an electron scanning microscope (SEM). Their chemical composition was analyzed via energy-dispersive X-ray (EDS) analysis. Their phase composition was tested via X-ray diffraction. The transmittance of the layers of lanthanum oxide was determined with the use of UV-Vis spectroscopy. The obtained layers of lanthanum oxide were characterized by a nanocrystalline microstructure and stable cubic structure. They also exhibited good transparency in both ultraviolet (UV) and visible (Vis) light.

## 1. Introduction

Lanthanum oxide (La_2_O_3_) is one of the most promising rare earth oxides. Due to its interesting properties, lanthanum oxide can be used in electronics, optoelectronics, and optics [1,2,3,4,5,6,7,8,9,10,11,12,13,14,15,16,17,18,19,20,21,22,23]. As a dopant, lanthanum oxide may affect the microstructure, structure, and properties of glass [24,25]. Lanthanum oxide is a catalyst [26,27]. La_2_O_3_ used as a dopant has a promotional influence on the performance of other catalysts [28,29,30,31]. It also influences the microstructure and mechanical properties of sintered WC-Co alloys [32]. Doping with lanthanum oxide can improve the resistance of aluminized coatings to high-temperature oxidation and corrosion [33]. It can also improve the corrosion resistance of alumina ceramics [34]. La_2_O_3_ added to metallic, as well as composite coatings, also allows for higher microhardness and better wear properties to be achieved [35,36,37,38,39,40,41,42,43]. These coatings also exhibit higher corrosion resistance [39,44]. In the case of metallic coatings, an increase in electrical conductivity is also possible [44].

Good results can be obtained by introducing an admixture of La_2_O_3_ to ceramic top coats in TBCs (Thermal Barrier Coatings) deposited via PVD (Physical Vapor Deposition), as well as APS (Atmospheric Plasma Spray) techniques [30,31,45,46,47,48,49,50,51,52,53,54,55,56]. YSZ (Yttria-stabilized Zirconia) doped with lanthanum oxide exhibits lower thermal conductivity than undoped YSZ [45,46,47,48,49]. The lifetime of these coatings can be longer than the YSZ top coat [50,51]. Its presence also affects their resistance to high-temperature oxidation [52,53,54]. La_2_Ce_2_O_7_ top coats deposited via APS techniques are characterized by lower thermal conductivity, a larger thermal expansion coefficient, and higher resistance to sintering than YSZ deposited via this method [55,56]. They also exhibit a longer lifetime and higher resistance to molten CMASs (calcium–magnesium–alumina–silicates) than YSZ coatings obtained using the same method [55,56]. Mullite top coats with lanthanum oxide for TBC indicate higher fracture toughness and hardness, as well as higher resistance to corrosion, spallation, and thermal shocks in comparison with mullite coatings without La_2_O_3_ [57]. In both cases, the top coats were deposited using the TAP (Transferred Arc Plasma) method [57].

Ceria-based electrolytes co-doped with lanthanum oxide for SOFCs (Solid Oxide Fuel Cells) exhibit a significant increase in their ionic conductivity in comparison with single-doped ceria electrolytes [31,58,59,60,61,62,63]. Ceria may be co-doped with La_2_O_3_ and Y_2_O_3_ [58], La_2_O_3_ and CaO [59], La_2_O_3_ and Sm_2_O_3_ [60], La_2_O_3_ and SrO [61], La_2_O_3_ and Dy_2_O_3_ [62], or La_2_O_3_ and In_2_O_3_ [63]. Co-doped ceria was obtained in the form of pellets via the sintering process at 1300–1500 °C [58,59,60,61,62,63]. However, the aim of the current research is to reduce the temperature for cell manufacturing, as well as its operation, to 600–800 °C. Moreover, it should be noted that SOFCs with tubular geometry are more advantageous than planar ones [64,65]. It seems that the solution to the above problems is the development of technology for the production of electrolytes, where these electrolytes would be in the form of non-porous and nanocrystalline thin layers deposited on tubular substrates in the above temperature range. The latest reports [65] show that, currently, thin-layer electrolytes for tubular SOFC are produced only with the use of “wet chemistry” methods, where after the deposition process, the obtained layers must be sintered at 1400–1500 °C. It is worth noting that research results included in works, e.g., [66,67,68], indicate that non-porous and nanocrystalline two-component electrolyte layers of CeO_2_-Sm_2_O_3_ [66], CeO_2_-Gd_2_O_3_ [67], CeO_2_-Y_2_O_3_ [68], and others can be synthesized on tubular substrates using the MOCVD technique at 600–800 °C. However, to obtain such two-component systems using this method, it is important to recognize the conditions for the synthesis of each component. For this reason, research on the synthesis of lanthanum oxide layers on tubes using MOCVD was undertaken in this study.

Thin lanthanum oxide layers have been obtained using techniques such as ALD (Atomic Layer Deposition) [1,8,10,11,12,21,22,23], PVD [2,4,5,6,15,18], MOCVD [3,7,9], spray pyrolysis [14,16], sol–gel [13], and spin coating [19]. However, they have been deposited only on planar substrates. It should be noted that the use of PVD techniques only makes layer deposition possible on planar substrates. In the case of sol–gel, spin coating, and other “wet chemistry” methods, as mentioned, the sintering of the deposited layers at high temperatures is necessary. MOCVD allows one to deposit layers even on complex-shaped substrates at low temperatures because the use of more reactive metalorganic reactants enables a decrease in the process temperature in comparison to conventional high-temperature CVD. However, the conditions suitable for film deposition on planar substrates cannot be transferred to substrates with other shapes, especially complex ones. If they were transferred, the obtained layers could be (locally) porous and vary in thickness.

The aim of this work was to obtain non-porous and nanocrystalline lanthanum oxide layers on the inner surfaces of tubular substrates using the MOCVD technique with the use of La(tmhd)_3_ (tris(2,2,6,6-tetramethyl-3,5-heptanedionato)lanthanum(III)) as the basic reactant. It was assumed that the synthesis process of lanthanum oxide layers would be carried out at 600–750 °C, and its other parameters would be determined based on the extended Gr_x_/Re_x_^2^ expression [69,70]. In this work, the synthesis conditions were established based on the extended Gr_x_/Re_x_^2^ criterion [69,70], where Gr—Grashof’s number, Re—Reynolds number, and x—the distance from the gas inflow point. This expression seems to be very important. It contains parameters omitted by other authors dealing with the CVD process. In particular, the parameters, such as the magnitude and gradient of static gas pressure, should be mentioned. Its effect on the reactant flow rate is very high. The thickness of the boundary, diffusion, and thermal layers are correlated with the reactant flow rate. The thickness of these layers is of great importance. It has a significant impact on the microstructure of synthesized layers, their uniformity in thickness, and their adhesion to the substrate. The curvature of the substrate surface may cause changes in the static gas pressure, which will affect the distribution of gas velocity in the boundary layer and the layer growth rate on convex and concave surfaces. The increasing gradient of the static gas pressure causes the gas flow rate to become faster [69,70].

It was expected that the synthesis process of La_2_O_3_ layers conducted at low values of this criterion should ensure the growth of non-porous layers on the inner surfaces of tubular substrates.

## 2. Materials and Methods

Lanthanum layers were manufactured via the MOCVD process from commercial La(tmhd)_3_ (98%, Alfa Aesar GmbH & Co KG, Karlsruhe, Germany). The carrier gases were pure argon (99.9999%) and air. Additionally, the oxygen contained in the air (as a source of oxygen) was also used to remove carbon from the growing layers. Carbon is a solid by-product of La(tmhd)_3_ pyrolysis. It is an impurity of the layers and may have an impact on their properties (e.g., it may be a reason for the deterioration of electrical properties of the deposited layers).

The quartz glass substrates were in the form of tubes with an internal diameter of 13 mm and a length of 25 mm. The wall thickness was 1 mm. Before the process of layer synthesis, the substrates were cleaned using distilled water and then ethyl alcohol (p.a.) in an ultrasonic washer.

The diagram of equipment applied for layer deposition is shown elsewhere [71].

Due to the fact that the shape of the substrate, various faults, and the roughness of the surface substrate have a significant impact on the gas flow conditions in the CVD reactor, it was assumed that the value of the extended Gr_x_/Re_x_^2^ expression [69,70] should be low. This should allow us to avoid turbulent gas flow and, as a consequence, prevent the homogeneous nucleation process during the growth of the synthesized layer. Taking into account the shape of the substrate and the possible roughness of its surface, it was assumed that the value of this criterion should be lower than 0.01. It was expected that the laminar gas flow in the CVD reactor could then be ensured. The process of the synthesis of lanthanum oxide layers was realized at 600–750 °C. La(tmhd)_3_ was evaporated at 170 to 200 °C. The magnitude of airflow amounted to 0.1–10 NL/h, and the magnitude of argon flow was 2 NL/h. The static gas pressure was changed in the range of 10 to 1.3 × 10^4^ Pa. The time of the deposition process amounted to 10–20 min.

The deposited layers of lanthanum were initially observed for the possibility of the occurrence of homogeneous nucleation during their growth. Due to the fact that no powders were noticed on their surfaces, the obtained samples were tested using a scanning electron microscope, SEM NANO NOVA 200, fabricated by FEI Europe Company (Eindhoven, The Netherlands), and an energy dispersive X-ray spectroscope (EDS) microanalyzer from EDAX EDS Company (Pleasanton, CA, USA). For these tests, the samples were broken into smaller fragments. Fragments of quartz glass with a La_2_O_3_ layer on its inner surfaces were intended for SEM observation. It was possible to see not only the layer surface but also the cross-section: substrate—lanthanum oxide layer. It was also possible to check the elemental composition of the layer (the average EDS analysis) and test changes in the elemental composition of this cross-section (the linear EDS analysis). The phase composition of the obtained samples was investigated using an X-ray diffractometer fabricated by Panalytical (Malvern, UK). The measurements were performed via GID (Grazing Incidence Diffraction). The radiation source was an X-ray tube with a linear focus and a Cu anode. The radiation beam was monochromatic (line K_α1_ = 1.5406 Å).

The transmittance of the substrates covered with La_2_O_3_ layers, as well as those without layers, was tested with the use of a UV-Vis Spectrophotometer, JASCO V630, fabricated by JASCO Deutschland GmbH (Pfungstadt, Germany). The samples (quartz glass tubes and quartz glass tubes with the obtained lanthanum oxide layers) were cut lengthwise in two parts for transmittance tests. The obtained halves of the samples were taken for testing.

## 3. Results and Discussion

**Figure 1:**
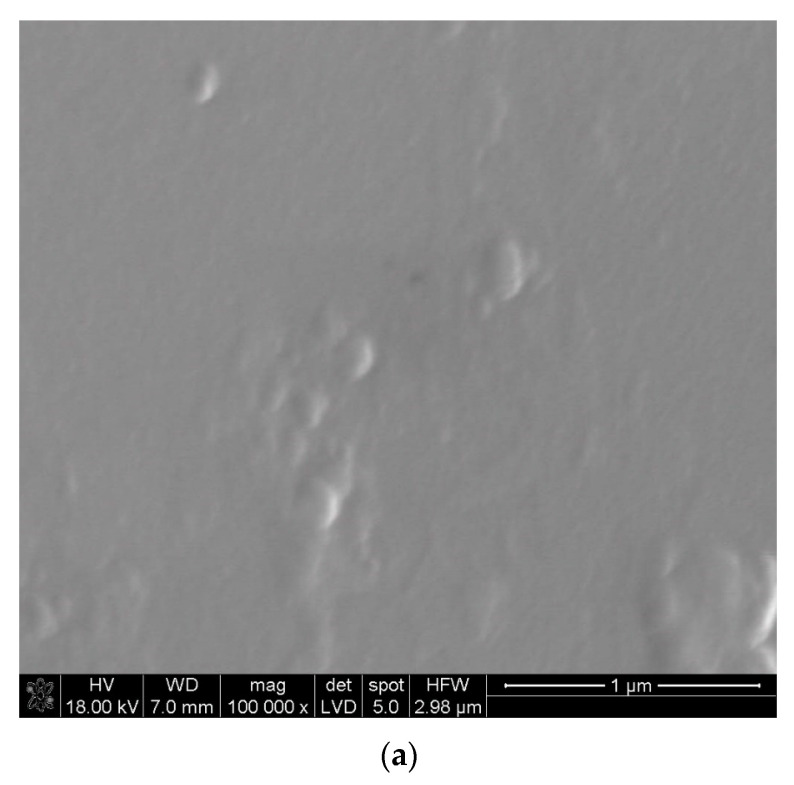

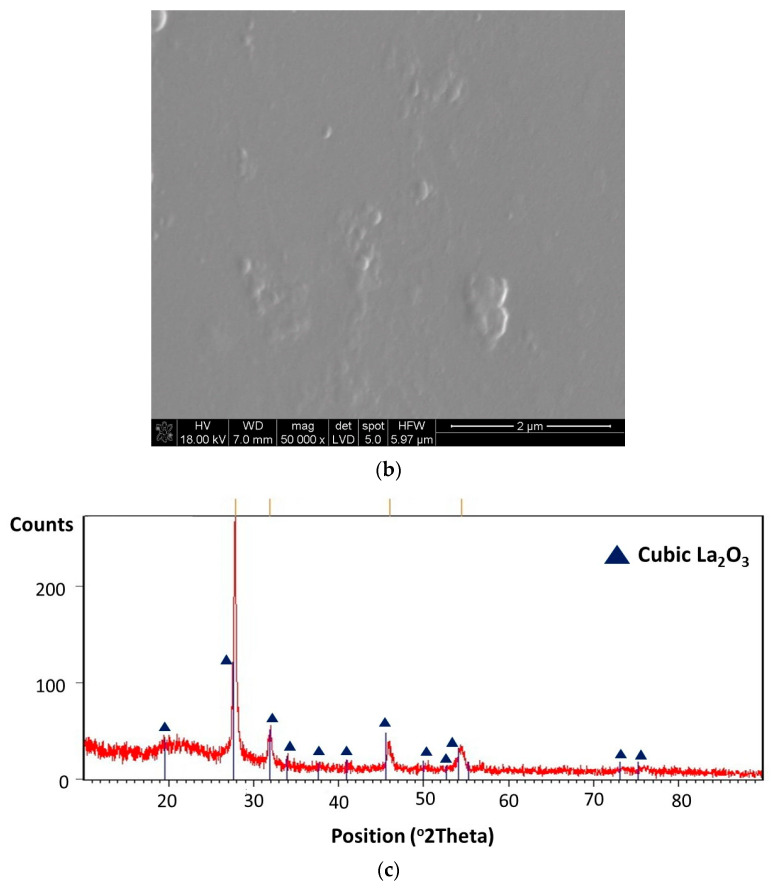
SEM microstructure of the La_2_O_3_ layer at different magnifications (**a**,**b**). Results of X-ray analysis of this layer (**c**). Layer deposition temperature: 600 °C. Synthesis time: 20 min. Evaporation temperature of La(tmhd)_3_: 185 °C.

The obtained layers of lanthanum oxide were glossy, with no traces of powders on their surfaces. The thickness of the deposited layers was estimated based on the interference colors [72]. The layer thickness was approximately 0.1–0.25 μm. SEM images of the microstructure of the layer deposited at 600 °C are presented in Figure 1a,b at different magnifications. La(tmhd)_3_ was evaporated at 185 °C, and the synthesis time was 20 min. From Figure 1a,b, it can be seen that there are no pores. And Figure 1c shows that the La_2_O_3_ layer is nanocrystalline with a cubic structure (JCPDS file No. 00-022-0369). The crystallite size estimated from Scherrer’s equation is in the range of 11–18 nm. It should be noted that quartz glass may contain different impurities, e.g., the grains of not completely melted sand. These fragments of the substrate surfaces are characterized by higher surface energy than the substrate surfaces without these defects.

Consequently, the processes of crystallization, as well as the merging of small crystallites and the formation of large aggregates, occur more easily in these places (Figure 1a,b).

**Figure 2:**
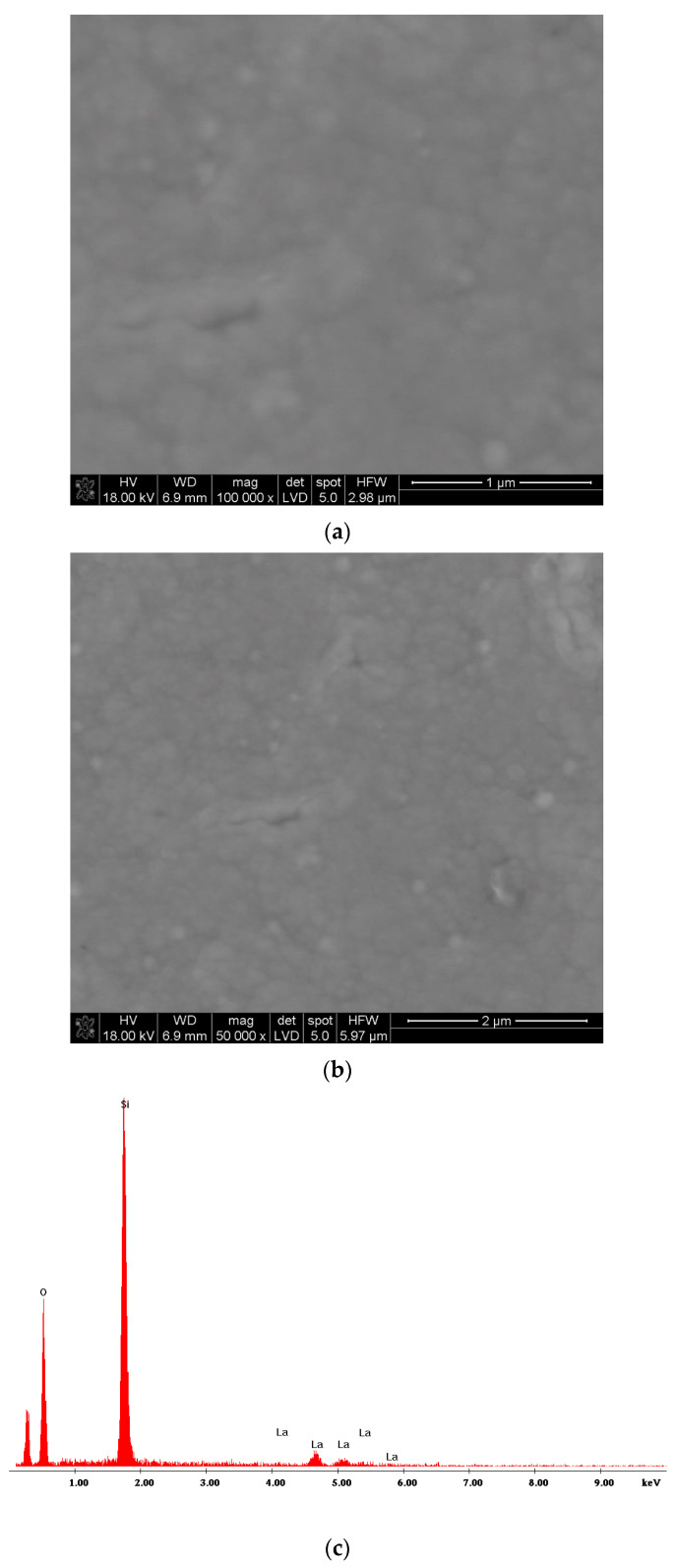
SEM microstructure of the La_2_O_3_ layer at different magnifications (**a**,**b**). EDS spectra of the sample (**c**). Layer deposition temperature: 650 °C. Synthesis time: 10 min. Evaporation temperature of La(tmhd)_3_: 195 °C.

**Figure 3:**
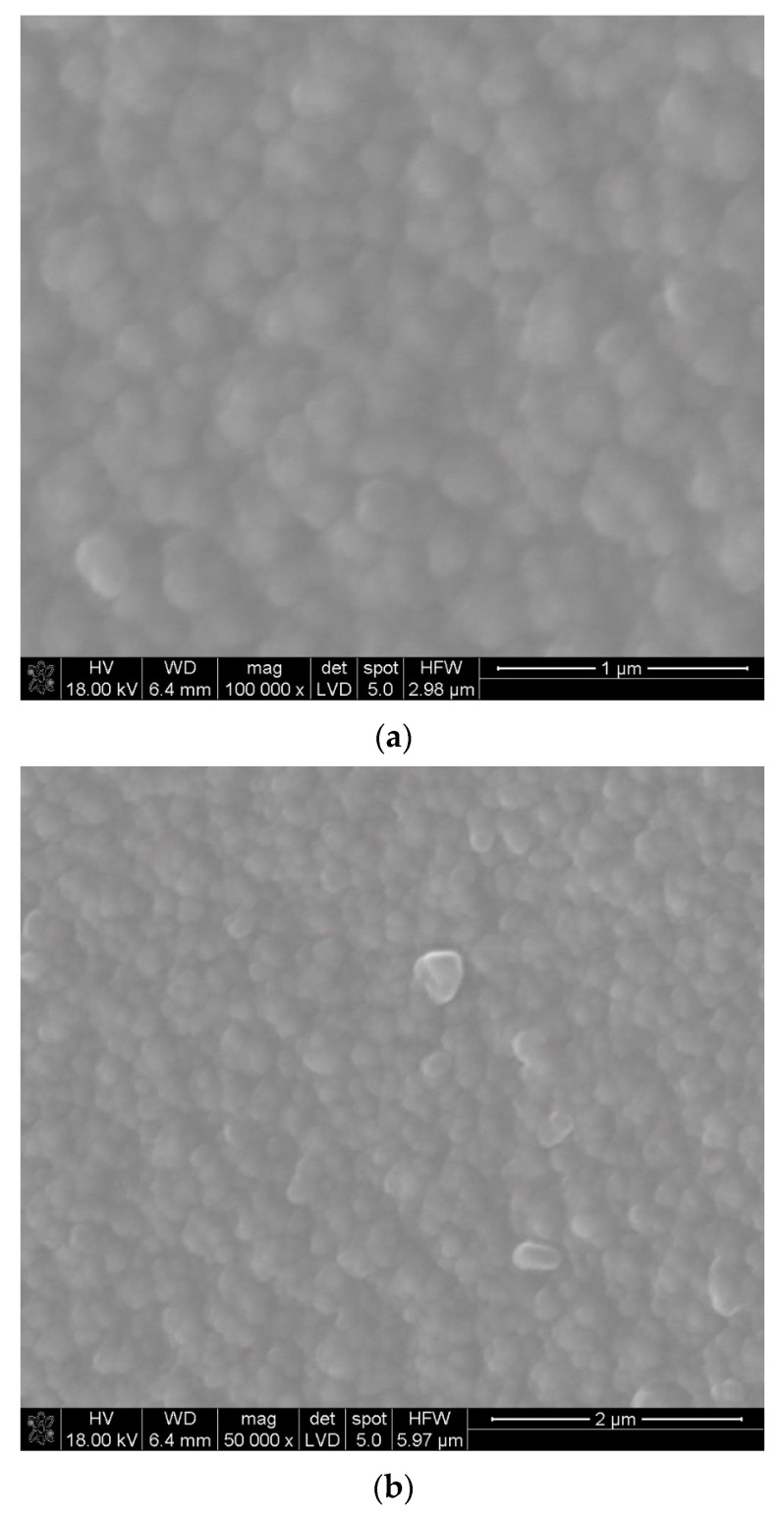

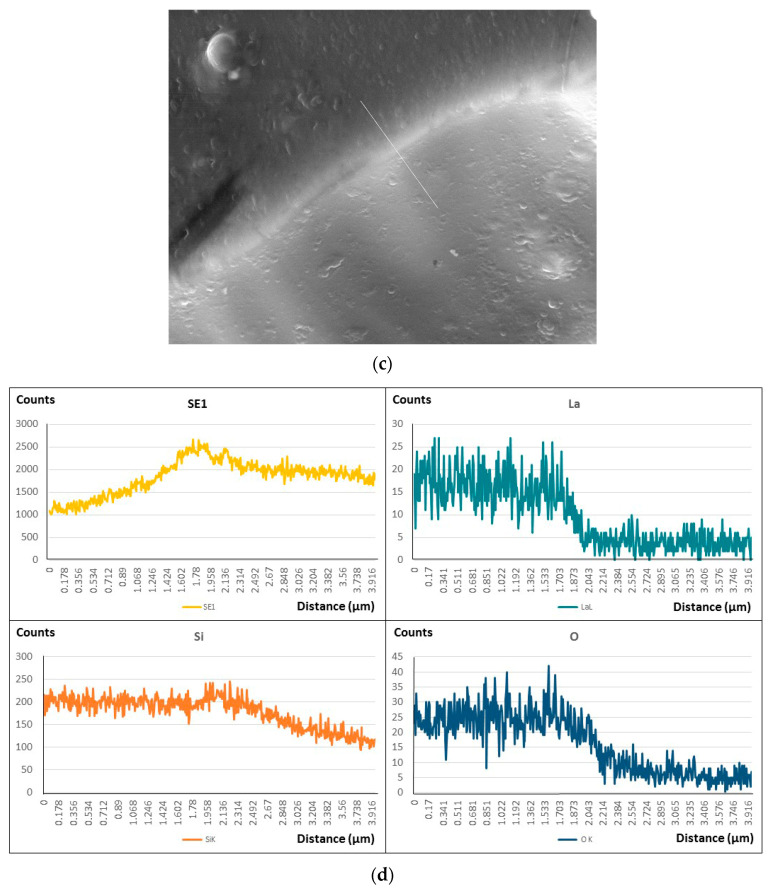
SEM microstructure of the La_2_O_3_ layer at different magnifications (**a**,**b**). Cross-section of the lanthanum oxide layer—quartz glass substrate (**b**) with linear EDS analysis along the marked line (**c**,**d**). Layer deposition temperature: 650 °C. Synthesis time: 15 min. Evaporation temperature of La(tmhd)_3_: 185 °C.

**Figure 4:**
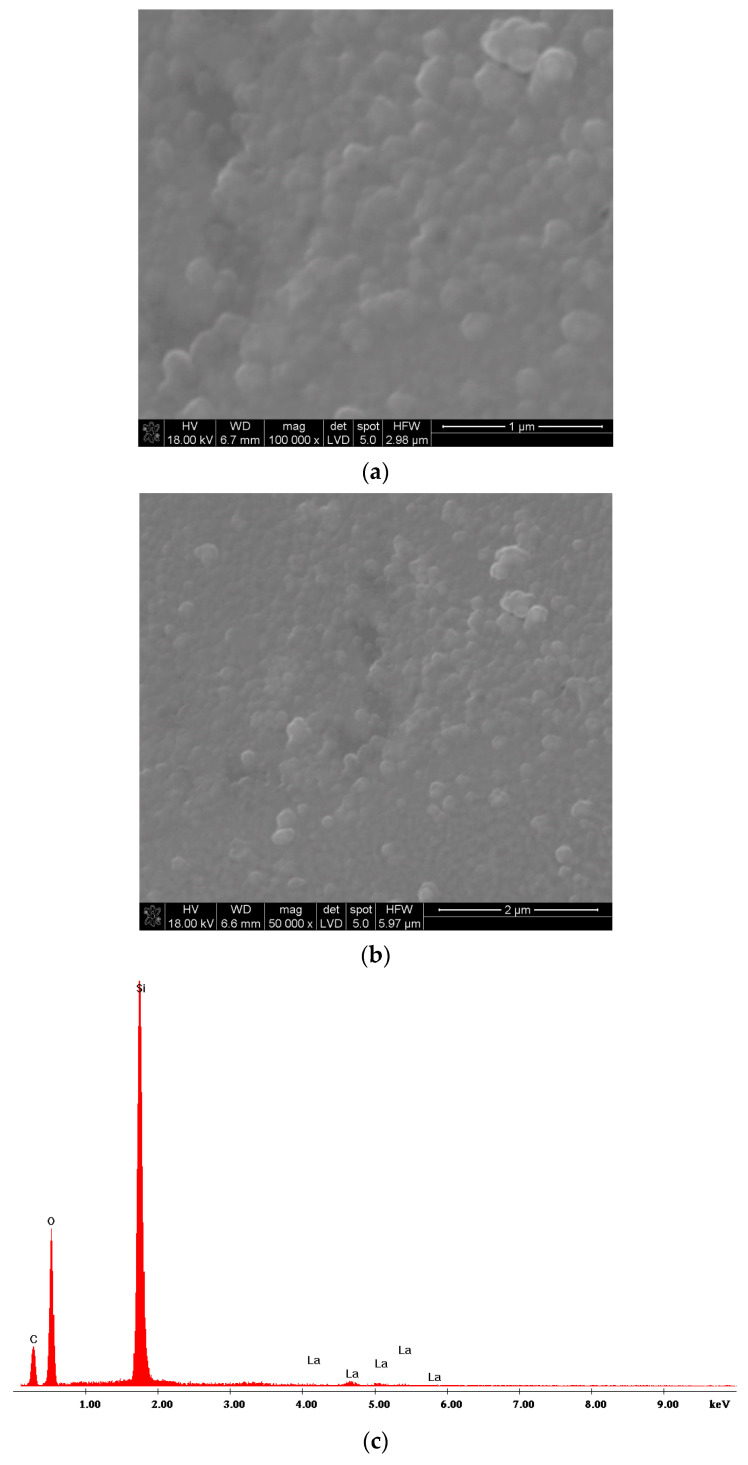
SEM microstructure of the La_2_O_3_ layer at different magnifications (**a**,**b**). EDS spectra of the sample (**c**). Layer deposition temperature: 650 °C. Synthesis time: 20 min. Evaporation temperature of La(tmhd)_3_: 185 °C.

Figure 2a,b, Figure 3a,b and Figure 4a,b present the results of SEM observations of lanthanum oxide layers synthesized at 650 °C. The synthesis time amounted to 10 min, 15 min, and 20 min, respectively. There were also different evaporation temperatures of La(tmhd)_3_. When the synthesis time was 10 min, the evaporation temperature of the reactant amounted to 195 °C (Figure 2a,b). Figure 2a shows the La_2_O_3_ layer microstructure, and Figure 2c shows the EDS spectra of this sample. As before, the layer is without pores (Figure 2a,b) and contains lanthanum (Figure 2c).

In the other two cases, the reactant was evaporated at 185 °C (Figure 3a,b and Figure 4a,b). An example of the microstructure of the La_2_O_3_ layer synthesized at 650 °C when the evaporation temperature of La(tmhd)_3_ was lower and amounted to 185 °C (the synthesis time was 15 min) is shown in Figure 3a,b. Figure 3c,d illustrate the results of linear EDS analysis along the cross-section: lanthanum oxide layer—quartz glass substrate. It can be seen that the obtained microstructure of the lanthanum oxide layer is similar to that presented in Figure 2a,b. Lanthanum is also present in the layer (Figure 3c,d).

For comparison, Figure 4a,b show the microstructure of the lanthanum oxide layer obtained at 650 °C at different magnifications. Its synthesis time was longer. It was 20 min. The evaporation temperature of the basic reactant was also the same as before (i.e., 185 °C). If the synthesis time is longer, the crystallites may become larger due to the recrystallization process. Heating time causes the growth of larger grains at the expense of smaller ones. In this case, the formation of large aggregates was observed. In spite of the deposition temperature being the same, the synthesis time was longer and the formed aggregates were larger (Figure 2a,b, Figure 3a,b and Figure 4a,b). The average EDS analysis (Figure 4c) confirms the presence of lanthanum in the layer. However, it should be noted that the evaporation temperature of La(tmhd)_3_ was lower (it was 185 °C) than in the case of the layer presented in Figure 2 (the deposition temperature was the same in both cases). This means that the concentration of the reactant in the carrier gases was lower. Hence, the layer growth rate was lower.

**Figure 5:**
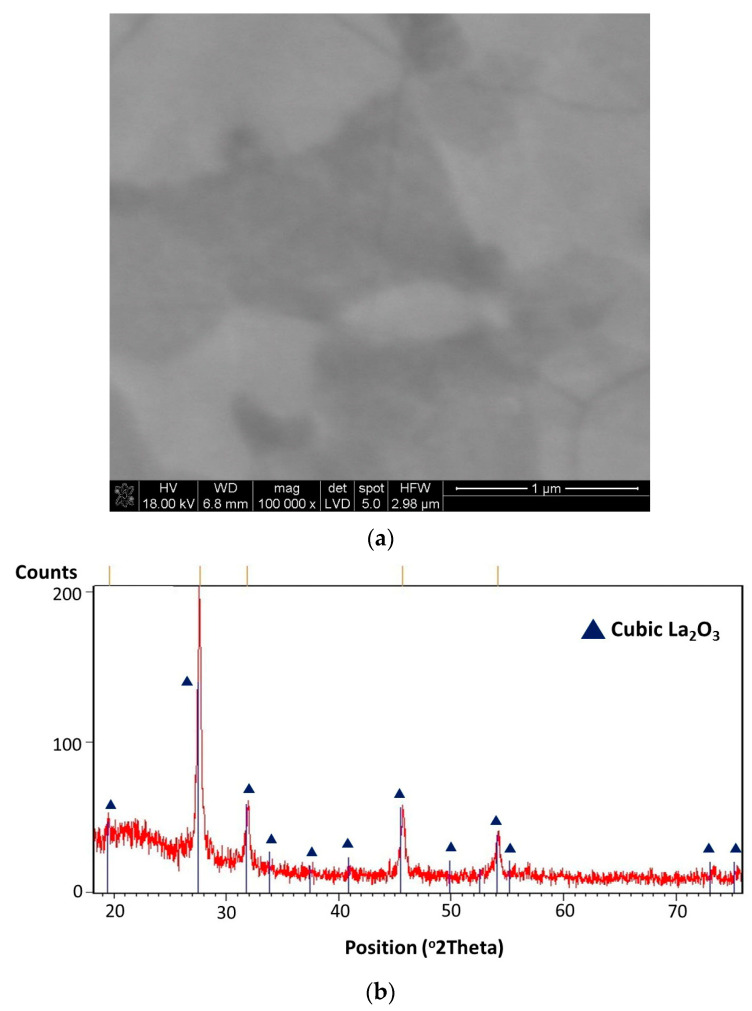
SEM microstructure of the La_2_O_3_ layer (**a**) and the results of the X-ray analysis of this layer (**b**). Layer deposition temperature: 750 °C. Synthesis time: 20 min. Evaporation temperature of La(tmhd)_3_: 195 °C.

A significant difference in the layer microstructure is visible in the case of the layer deposited at 750 °C for 20 min (Figure 5a). Both the high temperature of the process and its relatively long time contributed to the intensification of the formation of large aggregates, significantly larger than in the case of the layers synthesized at 650 °C (Figure 2b, Figure 3a,b and Figure 4a,b). The crystallization process of this layer seems to be more advanced than in the case of the layers growing at 600 °C (Figure 1a,b) and 650 °C (Figure 2a,b, Figure 3a,b and Figure 4a,b). However, the crystallite size is only slightly higher than the crystallite size of the layers deposited at 600 °C. The crystallite size is below 20 nm. It seems that if the synthesis time of the layers were much longer, there could be a greater difference in the crystallite size of the layers. Based on the X-ray analysis results (Figure 5b), it can also be concluded that this is a cubic lanthanum oxide (JCPDS file No. 00-022-0369). The layer is also non-porous (Figure 5a). It should also be noted that the evaporation temperature of La(tmhd)_3_ was higher than before. It was 195 °C in this case. Thus, there was a higher concentration of reactant in the gaseous reaction mixture and the layer growth rate was higher. Therefore, the obtained layer should also be thicker.

In the MOCVD process, when reactants are used in solid form, it is important to find the optimal temperature for their evaporation. On the one hand, it is important to increase the efficiency of the process, and on the other hand, it is important to prevent the decomposition process of reactants during their heating and the transport of their vapors over the heated substrate in the CVD reactor. It should also be noted that a high concentration of reactants, especially when the process temperature is high, favors reactions in the gas phase (the process of homogeneous nucleation). At high temperatures, molecules collide more easily. Consequently, reactions occur between them easily. In the case of a high reactant concentration, the problem can be solved by the use of a higher amount of diluent gases (e.g., argon), but the layer growth rate will then be lower. Turbulent gas flow also favors the process of homogeneous nucleation. In a turbulent gas flow, gas molecules also collide more easily with each other. Moreover, this has a very large impact on the heat transfer from the hot substrate to the cold gases. As a result, the temperature of gases in the entire volume of the reactor may increase, which will favor chemical reactions in the gas phase throughout the entire volume of the reactor. All irregularities, faults, and roughness of the substrate influence the nature of the gas flow. In the 1950s, Dryden [73] investigated the influence of substrate roughness on the value of the Re criterion (Reynolds number, Re_crit._). He stated that this number could be up to 200 if the substrate surface was very rough. According to Kwatera [70], the presence of any type of unevenness of the substrate and/or the heating of gases from the hot substrate may lead to a reduction in this number value, even to six. When the deposition process is realized on the substrate, even with significant roughness, it is possible to completely eliminate the homogeneous nucleation process and obtain a solid product only on the substrate. This is possible when the parameters of the deposition process are adjusted so that the value of extended Gr_x_/Re_x_^2^ expression is low. For this reason, lanthanum oxide layers were deposited on the inner surfaces of tubular substrates under optimal conditions, which should ensure that the value of this criterion is lower than 0.01.

**Figure 6:**
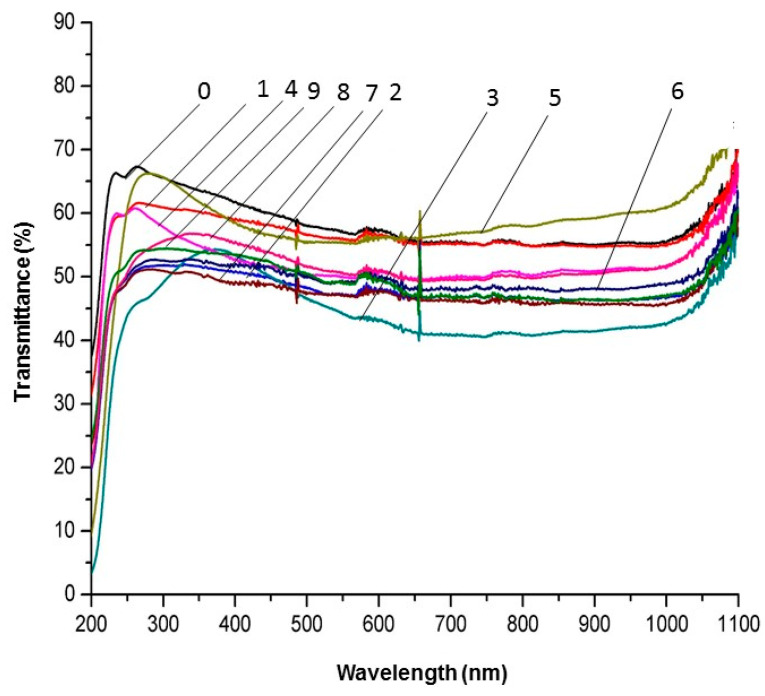
Transmittance of quartz glass covered with La_2_O_3_ layers under different conditions and without layers. 0—uncoated glass; 1—glass coated with La_2_O_3_ at 650 °C, evaporation temperature of La(tmhd)_3_: 170 °C, and deposition time: 20 min; 2—glass coated with La_2_O_3_ at 650 °C, evaporation temperature of La(tmhd)_3_: 185 °C, and deposition time: 20 min; 3—glass coated with La_2_O_3_ at 650 °C, evaporation temperature of La(tmhd)_3_: 185 °C, and deposition time: 15 min; 4—glass coated with La_2_O_3_ at 650 °C, evaporation temperature of La(tmhd)_3_: 190 °C, and deposition time: 20 min; 5—glass coated with La_2_O_3_ at 650 °C, evaporation temperature of La(tmhd)_3_: 195 °C, and deposition time: 10 min; 6—glass coated with La_2_O_3_ at 750 °C, evaporation temperature of La(tmhd)_3_: 185 °C, and deposition time: 20 min; 7—glass coated with La_2_O_3_ at 750 °C, evaporation temperature of La(tmhd)_3_: 195 °C, and deposition time: 20 min; 8—glass coated with La_2_O_3_ at 750 °C, evaporation temperature of La(tmhd)_3_: 195 °C, and deposition time: 15 min; 9—glass coated with La_2_O_3_ at 650 °C, evaporation temperature of La(tmhd)_3_: 190 °C, and deposition time: 20 min.

The quartz glass covered with lanthanum oxide layers and without layers was tested using a UV-Vis spectrophotometer. The results of these measurements are illustrated in Figure 6. Quartz glass covered with La_2_O_3_ layers under different conditions exhibits good transmittance in visible light. If homogeneous nucleation was present during the process of layer growth, then the obtained layers would be porous and their transmittance would be significantly reduced. They would be visually mat. The synthesis process of lanthanum oxide layers was realized with low values of extended Gr_x_/Re_x_^2^ expression. As mentioned, this should ensure laminar gas flow in the CVD reactor in spite of the tubular shape of the substrate and probable surface roughness resulting from the glass production process. As a consequence, this prevents the occurrence of homogeneous nucleation during the growth of the deposited layers. The results of SEM observations (Figure 1a,b, Figure 2a,b, Figure 3a,b, Figure 4a,b and Figure 5a) and UV-Vis transparency tests of the deposited lanthanum oxide layers (Figure 6) allow us to conclude that this process did not occur during layer growth. A slight reduction in the transmittance of quartz glass covered with lanthanum oxide layers may be caused by more or less advanced crystallization of the obtained layers.

A visible decrease in the transmittance of uncoated, as well as coated, glass is probably caused by the shape of the samples. As mentioned, the tubes were cut lengthwise and the obtained halves of the tubes were used for measurement. Their curved shape could have influenced the measurement results. Hence, the transparency is reduced even in the case of uncoated quartz glass.

## 4. Conclusions

Lanthanum oxide layers were obtained on the inner surfaces of tubular substrates using the MOCVD method at 600–750 °C. La(tmhd)_3_ was evaporated in the temperature range of 170–200 °C. La_2_O_3_ layers exhibited a stable cubic structure. They were nanocrystalline and without the presence of pores. The crystallite size was between 11 and 20 nm. An increase in the layer synthesis temperature from 600 to 750 °C with the same synthesis time caused only a slight increase in the crystallite size. An increase in the synthesis temperature and an extension of the synthesis time resulted in the merging of small crystallites into large aggregates. This process was particularly intense in the case of the layers deposited at 750 °C.

The use of low values of extended Gr_x_/Re_x_^2^ criterion (i.e., below 0.01) favors the growth of non-porous layers on the inner surfaces of tubular substrates. Low values of this expression allowed us to ensure laminar gas flow in the CVD reactor and obtain layers without pores on tubular substrates. Transmittance of quartz glass covered with La_2_O_3_ layers was only slightly reduced in comparison to uncoated glass due to the fact that the deposited layers of lanthanum oxide were crystalline.

## Data Availability

The original contributions presented in the study are included in the article, further inquiries can be directed to the corresponding author.
